# Insulin therapy in patients with cystic fibrosis in the pre-diabetes stage: a systematic review

**DOI:** 10.1016/j.rppede.2016.03.006

**Published:** 2016

**Authors:** Mariana Zorrón Mei Hsia Pu, Flávia Corrêa Christensen-Adad, Aline Cristina Gonçalves, Walter José Minicucci, José Dirceu Ribeiro, Antonio Fernando Ribeiro

**Affiliations:** a Faculdade de Ciências Médicas, Universidade de Campinas (Unicamp), Campinas, SP, Brazil

**Keywords:** Cystic fibrosis, Insulin, Diabetes mellitus

## Abstract

**Objective:**

To elucidate whether insulin is effective or not in patients with cystic fibrosis before the diabetes mellitus phase.

**Data source:**

The study was performed according to the Prisma method between August and September 2014, using the PubMed, Embase, Lilacs and SciELO databases. Prospective studies published in English, Portuguese and Spanish from 2002 to 2014, evaluating the effect of insulin on weight parameters, body mass index and pulmonary function in patients with cystic fibrosis, with a mean age of 17.37 years before the diabetes mellitus phase were included.

**Data synthesis:**

Eight articles were identified that included 180 patients undergoing insulin use. Sample size ranged from 4 to 54 patients, with a mean age ranging from 12.4 to 28 years. The type of follow-up, time of insulin use, the dose and implementation schedule were very heterogeneous between studies.

**Conclusions:**

There are theoretical reasons to believe that insulin has a beneficial effect in the studied population. The different methods and populations assessed in the studies do not allow us to state whether early insulin therapy should or should not be carried out in patients with cystic fibrosis prior to the diagnosis of diabetes. Therefore, studies with larger samples and insulin use standardization are required.

## Resumo

**Objetivo:**

Elucidar se a insulina é eficaz ou não em pacientes com fibrose cística antes da fase de diabetes.

**Fontes de dados:**

O estudo foi feito de acordo com o método Prisma entre agosto e setembro de 2014, nas bases de dados PubMed, Embase, Lilacs e SciELO. Foram incluídos estudos prospectivos publicados em inglês, português e espanhol de 2002 a 2014 que avaliaram o efeito da insulina nos parâmetros peso, índice de massa corporal e função pulmonar, em pacientes com fibrose cística, com média de 17,37 anos, antes da fase de diabetes.

**Síntese dos dados:**

Foram identificados oito artigos que incluíram 180 indivíduos submetidos ao uso de insulina. O tamanho das amostras variou de 4 a 54 pacientes, idade média entre 12,4 e 28 anos. O tipo de acompanhamento, o tempo de uso de insulina, a dose e o cronograma de implementação foram muito heterogêneos entre os estudos.

**Conclusões:**

Existem razões teóricas para se acreditar que a insulina tenha um efeito benéfico na população estudada. Os diferentes métodos e populações encontrados não permitem afirmar se a terapia precoce com insulina deve ou não ser feita em pacientes com fibrose cística previamente ao diagnóstico de diabetes. Portanto, são necessários estudos com amostras maiores e uniformidade de uso da insulina.

## Introduction

Cystic fibrosis-related diabetes (CFRD) is the most common comorbidity in patients with cystic fibrosis (CF) and affects 20% of adolescents and 40-50% of adults with CF.^1^

Glucose disorders in CF patients typically begin with an intermittent postprandial hyperglycemia, followed by oral glucose intolerance without fasting hyperglycemia and finally diabetes with fasting hyperglycemia.^2^^,^ 3

Insulin deficiency and resulting hyperglycemia affect lung disease.^3^^-^ 5 Insulin is a hormone with anabolic effects and its deficiency may have a negative clinical impact on patients considered "prediabetic".^6^ Increased serum glucose levels (≥144mg/dL) may have an adverse effect on lung function. Furthermore, increased glucose in the bronchial tree favors the growth of respiratory pathogens.^5^ There is still a loss of lean body mass due to the catabolic state caused by insulin deficiency, which leads to a consumption of fat and proteins and also affects pulmonary function.^7^

Therefore, insulin deficiency promotes a clinical deterioration in this population and not only an abnormal glucose metabolism, which may be enhanced by early intervention with insulin.^6^ Both diabetes and glucose intolerance reduce the life expectancy of CF patients; insulin is the only treatment that improves clinical outcomes.^8^ Early treatment with insulin may reduce the morbidity and mortality of the underlying disease.^9^^,^ 10

Moreover, CF patients' classification using the oral glucose tolerance test (OGTT) in intolerant and diabetic patients is based on criteria derived from epidemiological studies in non-CF subjects, it raises doubts whether these conventional diagnostic limits would be appropriate or relevant for CF patients.^11^ Thus, the use of conventional glucose evaluation tests in the CF population could underestimate the number of patients with abnormal glucose metabolism, and, consequently, this group could benefit from early intervention with insulin, in glucose levels below those considered abnormal in populations without cystic fibrosis.^12^

To our knowledge, there is no systematic review of early initiation of insulin therapy in CF patients. Therefore, the aim of this study was to identify the effects of this intervention and contribute to clinical practice and future studies.

## Method

The search process was developed according to the Prisma method (Preferred Reporting Items for Systematic Reviews and Meta-Analyses).^13^ The search was conducted between August and September 2014 in the following electronic databases: PubMed, Lilacs, SciELO, and Excerpta Medica Database (Embase).

The following terms and descriptors (Medical Subjects Headings - MeSH) were used for the search: 'cystic fibrosis', 'early insulin', 'insulin', 'body mass index', 'impaired glucose tolerance', and 'therapy'; in combinations: 'cystic fibrosis and early insulin', 'cystic fibrosis and insulin and body mass index', 'cystic fibrosis and early insulin', 'cystic fibrosis and insulin and body mass index', 'impaired glucose tolerance and cystic fibrosis and insulin and therapy'.

Studies published between 2002 and 2014 were identified through electronic search by two independent reviewers who evaluated the titles and abstracts of articles. References of selected articles were also reviewed in order to identify studies not found in the surveyed bases. Discrepancies between reviewers were discussed and resolved by consensus. The date of the first search was August 28, 2014, and the last, September 22, 2014.

Inclusion criteria were: (I) original articles; (II) prospective studies; (III) articles in English, Spanish or Portuguese; (IV) cystic fibrosis diagnosis; (V) glucose disorders; (VI) insulin use (regardless of type, dose, or implementation schedule); (VII) evaluation of the results in clinical parameters (weight or height or body mass index and pulmonary function). Glucose disorder was considered as an OGTT non-characterized as diabetes by the American Diabetes Association (ADA) criteria and OGTT glucose values above 140mg/dL at any time, except at baseline and 120min; or postprandial glucose random or above 200mg/dL; or impaired glucose tolerance (IGT) diagnosis by ADA criteria.^14^

Exclusion criteria were: (I) non-original articles, such as letters, conference proceedings and editorials; (II) studies evaluated only CFRD without other types of disorders of glucose.

The extracted data were: study design; sample size; population characteristics; follow-up time; type of insulin therapy (including dose and regime used); and effects on weight, body mass index, and lung function.

## Results

**Figure 1:**
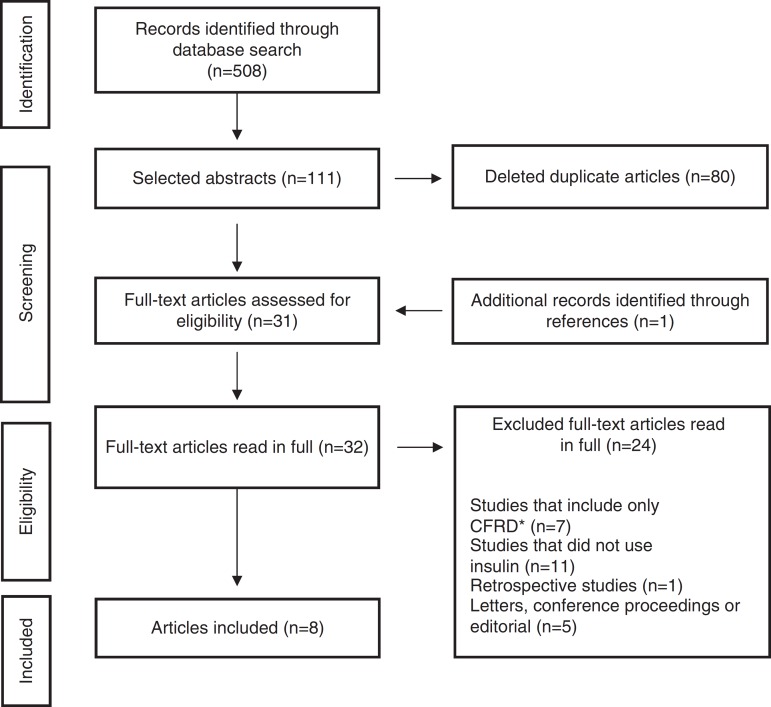
Flowchart of study identification with inclusions and exclusions (cystic fibrosis-related diabetes - *CFRD).

The initial search identified 508 articles, of which 111 were selected based on titles and abstracts. References of selected papers were also reviewed and an additional study was included. Of these, 80 were identified as duplicates and removed; thus, 32 articles were read in full, of which 24 were excluded by the exclusion criteria. The final selection consisted of eight items (Fig. 1). Characteristics of the study results are summarized in Table 1.

**Table 1:** Characteristics of included studies.

	Population			Intervention	Results	
	Sample characteristics	Mean follow-up (months)	Mean age (years) (min-max)	Insulin type and average dose	BMI	FEV1
Dobson et al.^6^	Normal OGTT glucose and postprandial >200mg/dL	3	20.25 (15-23)	NPH 6-8UI/d ou UR 5UI/d+7UI/d (70/30)	NA	NS^g^
Case report (n=4)						
Bizzarri et al.^20^	IGT	16.8	18.2 (9.2-27.8)	Glargine 0.3UI/kg/d	SI	SI
Clinical uncontrolled trial (n=6)					*p*=0.026	*p*=0.027
Mozzillo et al.^18^	2: CFRDFH+	12	12.4 (2.6-19)	Glargine 0.23UI/kg/d	SI^d^	SI
Clinical uncontrolled trial (n=22)	7: CFRDFH-					
			9: IGT		*p* =0.017	*p* =0.01
	4^a^					
Moran et al.^15^	23: CFRDFH-	12	28±9	Aspart 0.5UI: 15gCHO	SI^e^	NS
Randomized controlled trial (n=30)	7: severe IGT ^b^				*p* =0.02	
Koloušková et al.^17^	17: CFRDFH- 11: IGT	36	15.3^c^ (11.1-20.6)	NPH 0.12-0.25UI/kg/d	SI^f^	SI^h^
Randomized controlled trial (n=28)					*p* <0.05	*p* =0.03
Drummond et al.^12^	24: CFRD	69.36	27.64 (16-52)	UR, premixed, basal and basal-bolus	NA	NS
Clinical uncontrolled trial (n=54)	18: IGT					
	12: NGT					
Hameed et al.^19^	2: CFRDFH+	9.6	12.5 (7.2-18.1)	Detemir 0.13UI/kg/d	NA	SI
Clinical uncontrolled trial (n=18)	4: CFRDFH-					
	6: IGT					*p* =0.007
	6: NGT					
Minicucci et al.^16^	IGT	18	18 (11-53)	Glargine 0.1-0.15UI/kg/d	NS	NS
Randomized controlled multicenter clinical trial (n=18)						

CFRDFH+, CFRD with fasting hyperglycemia; CFRDFH-, CFRD without fasting hyperglycemia; NGT, normal glucose tolerance; CHO, carbohydrate; FEV1, forced expiratory volume in one second; UR, ultrafast insulin; NA, data not evaluated; NS, not significant; SI, significant increase.

a One or more of OGTT values >140mg/dL (between T30 and T90).

b OGTT >200mg/dL at any time and 180-199mg/dL 120min.

c Median.

d Only in group with initial Z-score <-1.

e Only CFRDFH- group.

f Only insulin deficiency group.

g Increase suggested, but without statistical evaluation.

h Compared with control group (there was no statistical difference intragroup).

Sample size of the included studies ranged from 4 to 54 patients, with mean age from 12.4 to 28 years. Investigators and subjects were not blind to the treatment assignment in any of the studies.

Type of follow-up, insulin time, dose, and implementation schedule were very heterogeneous, which can be seen in Table 1 . Three studies used control groups to compare the effects of insulin. Moran et al.^15^ selected corresponding controls who underwent other types of intervention (repaglinide or placebo), while Minicucci et al.^16^ used controls with IGT and Koloušková et al.^17^ used controls with normal OGTT by ADA criteria (NGT). In these last two studies, controls did not undergo pharmacological interventions.

Inclusion criteria for studies were very heterogeneous. Mozzillo et al.^18^ used the following inclusion criteria: no use of systemic corticosteroids and no exacerbation of lung disease. Minicucci et al.^16^ included patients with at least one of the following conditions: (I) body mass index BMI<10th percentile (p^10^); (II) loss of one BMI percentile for age and sex in the previous year; (III) forced expiratory volume in one second (FEV1) ≤80% of predicted; and (IV) decreased FEV1≥10% in the previous year. Lung function deterioration and weight loss were also criteria for inclusion of subjects in the study by Dobson et al.^6^ In contrast, Moran et al.^15^ chose to intervene in a more clinically stable group of patients and used the following inclusion criteria: (I) end of linear growth; (II) weight stability in the last three months; (III) absence of acute infection in the last two months. Exclusion criteria for this study were: (I) use of oral or intravenous corticosteroids in the last six months; (II) fasting hyperglycemia in the previous year; (III) liver dysfunction; (IV) pregnancy. Early insulin deficiency, diagnosed by intravenous glucose tolerance test (IVGTT) and/or high levels of glucose in OGTT, was used as inclusion criteria in the studies by Koloušková et al.^17^ and Hameed et al.^19^

Five studies evaluated the effects of insulin in BMI of CF patients.^15^^-^ 18 ^,^ 20 Bizzarri et al.,^20^ Moran et al.,^15^ and Koloušková et al.^17^ demonstrated a significant increase in BMI after insulin intervention. Mozillo et al.^18^ found a significant increase in BMI only in patients with initial BMI Z-score <-1. Although Moran et al.^15^ identify improvements in BMI in the group as a whole, in particular IGT group they did not notice a significant increase in this parameter. Dobson et al.,^6^ Drummond et al.,^12^ and Hameed et al.^19^ chose to assess body weight. Hameed et al.^19^ and Drummond et al.^12^ found significant weight gain after insulin intervention, while Dobson et al.^6^ suggested this trend, as data were not statistically evaluated.

FEV1 was the only clinical parameter assessed by all studies. Bizzarri et al.,^20^ Mozzillo et al.,^18^ and Hameed et al.^19^ found a significant increase in FEV1 after the use of insulin. Koloušková et al.^17^ found that, at the end of follow-up, intervention group had higher FEV1 compared to control group. Dobson et al.^6^ showed an apparent increase in this parameter with the use of insulin, as it was only a case report. In studies by Moran et al.,^15^ Drummond et al.^12^ and Minicucci et al.,^16^ FEV1 remained unchanged after the intervention. But Drummond et al.^12^ evaluated separately only patients diagnosed with IGT and found a significant reduction in FEV1 decline rate in patients using insulin. Hameed et al.^19^ evaluated separately only the early insulin-deficient patients (excluding patients with CFRD) and also found in this group a significant increase in FEV1. Moran et al.^15^ reported a lower apparent decline in FEV1 in patients using insulin compared to placebo, but this stability was not statistically significant.

Mozzillo et al.^18^ found a reduced number of pulmonary exacerbations (as compared to the previous year), while Bizzarri et al.^20^ found no changes in the number of hospitalizations for exacerbations. The four patients evaluated by Dobson et al.^6^ showed an increase in forced vital capacity (FVC) with the use of insulin. Hameed et al.^19^ found a significant improvement in FVC after the intervention.

In the results found by Bizzarri et al.,^20^ there was no significant change in the levels of glycosylated hemoglobin (HbA1c) after insulin, whereas the group of patients evaluated by Minicucci et al.^16^ showed a significant reduction in HbA1c with the use of insulin.

Frequent episodes of hypoglycaemia were reported only by Drummond et al.^12^ In the other studies cited in this review, the adverse effects of insulin therapy were infrequent and well tolerated.

## Discussion

There are few published papers on the use of insulin in patients with cystic fibrosis prior to overt diabetes. Most are limited to a single center and mainly to adults. To our knowledge, this is the first systematic review to examine the benefits and risks of insulin use in CF patients before the diagnosis of diabetes.

Glucose intolerance indicates the presence of insulin deficiency, which leads to a protein consumption and negative clinical/nutritional impact. Therefore, early treatment of insulin can have a positive effect in CF patients in the prediabetic phase.^20^

The study results make sense when considering the pathophysiology of the evolution to CFRD. Initially, there is an insulin deficiency that generates a protein catabolism and glycemic excursions, with consequent difficulty gaining and maintaining weight and worsening of lung function. Therefore, the introduction of insulin at this stage would likely prevent the catabolic effects of insulin deficiency.

Current data are clear about the insulin treatment for patients with CFRD with or without fasting hyperglycemia,^14^ but there are no consistent results to determine whether this treatment should also be used for those with other glucose disorders, as it is not well defined what are glucose disorders in this specific population. Moreover, there is doubt whether the cut-off values for the diagnosis of CFRD and IGT are valid for CF because they are based on population without the disease.

A negative impact of the prediabetic phase is described in nutritional status and pulmonary function of CF patients,^6^^,^ 21 it suggests that insulin should be started before the diagnosis of CFRD by the current methods available, as insulin has anabolic effects and these patients have few side effects (hypoglycemia). The only study reporting frequent episodes of hypoglycemia was the study by Drummond et al.,^12^ but they included several insulin therapy regimens in patients with CFRD, IGT, and NGT, and there was no description of the insulin type or dose instruction for each group, which may be related to the difference in the frequency of hypoglycemia seen between studies.

Although most studies have a small sample size and used more than one type of insulin, only Moran et al.^15^ and Minicucci et al.^16^ reported no positive effects with early insulin therapy, but these studies have some peculiarities described below, suggesting that early initiation of insulin therapy in CF patients could be beneficial.

Minicucci et al.^16^ reported no clinical improvement with the use of glargine in CF patients with IGT (ADA criteria). The authors assumed that participation in the study made patients more aware of their change in glucose metabolism, leading to better nutritional behavior. Most other studies^6^^,^ 17 ^-^ 20 showed positive results, but the insulin doses used were higher.

Mozzillo et al.^18^ found that after 12 months of therapy with insulin, BMI curve (Centers for Disease Control - CDC) improved in patients with baseline Z-score below -1, which is in agreement with the study by Koloušková et al.^17^ that also reported improvement in BMI, regardless of the baseline Z-score.

Koloušková et al.^17^ demonstrated that insulin administration has positive effects on lean body mass due to protein catabolism reversal. However, control group also showed a tendency towards that improvement, probably due to better nutritional orientation, as in both groups there was a recommendation for increased caloric intake up to 120-150% of the daily needs. According to the authors, the results support the concept that insulin deficiency, assessed in this study by using IVGTT and OGTT, leads to clinical deterioration in CF patients and that early initiation of insulin therapy could be recommended earlier than it is currently accepted (CFRD).

Dobson et al.,^6^ Bizzarri et al.,^20^ and Hameed et al.^19^ found weight improvement in patients using insulin.

Moran et al.^15^ reported that insulin reverses weight loss in patients with CFRD without fasting hyperglycemia, but not in patients with severe IGT. However, the study population was in adulthood and there seems to be a better response when these individuals are in childhood and adolescence. Moreover, the group in question had severe IGT (OGTT≥200mg/dL at any time and 120min between 180 and 199mg/dL), and in other studies, patients were previously selected in severe IGT phase, which may explain the differences found in the results.

Bizarri et al.^20^ found improved FEV1 with no reduction in pulmonary exacerbations. Mozzillo et al.^18^ reported increased FEV1 and reduced pulmonary exacerbations with the use of insulin. Hameed et al.^19^ demonstrated a fall in FEV1 before starting treatment and improvement after the introduction of insulin. Koloušková et al.^17^ and Drummond et al.^12^ assessed FEV1 compared to untreated subjects and identified a decline in lung function in control group, which was not seen in insulin-treated group. Moran et al.^15^ and Minicucci et al.^16^ found no improvement in FEV1 with early use of insulin. However, in the study by Minicucci et al.^16^ there was a 10% decrease in FEV1 in the year prior to the intervention, which may be a bias because even CFRD patients have no such decline in this parameter. Koloušková et al.^17^ and Drummond et al.^12^ , who had a more consistent number of cases, found that FEV1 was lower in the untreated group compared to those treated with insulin, which confirms the results described by Dobson et al.^6^ Bizarri et al.^20^ , and Hameed et al.^19^

Dobson et al.^6^ suggest an improvement in pulmonary function (FEV1 and FVC) with the use of insulin. However, their sample size was small (n=4), selected by convenience, and had no control; the reevaluation occurred in a short period of time (3 months) without insulin standardization (more than one type of insulin was used) and there was no statistical analysis, probably due to the sample size.

Hameed et al.^19^ assessed height separately and found no differences after insulin therapy initiation. This is probably due to the result seen in another study by Bizzarri et al.^22^ They suggest that in the development of CFRD there is already substantial and irreversible impairment of height because most patients with CF develop diabetes at puberty, the same time that the growth spurt occurs.^22^

The evidence, favorable or unfavorable, to the use of insulin before overt diabetes in CF patients remains inconclusive, with little knowledge about long-term results. There are few prospective studies on the use of insulin before overt diabetes in patients with CF and they include population with different types of glucose disorders, without age group delimitation, follow-up time, and insulin type, dosage, and implementation schedule. Only two studies^15^^,^ 16 were multicenter and only three were controlled.^15^^-^ 17 Moreover, it was not possible to assess the effect of important variables, such as nutritional routine, and a standard definition of glycemic disorders.

When analyzing the results of studies regarding the anabolic effects of insulin, there are theoretical reasons to believe that insulin has a beneficial effect on the population studied. However, the addition of a treatment for diabetes in a multiple drug treatment regimen is complicated, which makes this decision even more controversial.

Multicenter trials in randomized pediatric patients, with adequate nutritional support, type and standard doses of insulin (enough to promote anabolism), are needed to determine if treatment is justified or not. Keep in mind that placebo-controlled trials are difficult to perform due to the fact that insulin is an injectable medicine.

## Conclusion

The different methods and case series used in the studies do not allow affirming that early insulin therapy should be applied in patients with CF and glucose disorders. To this end, studies with larger samples, diet standardization, age group, and uniformity of insulin use are needed.
